# The nucleus does not significantly affect the migratory trajectories of amoeba in two-dimensional environments

**DOI:** 10.1038/s41598-019-52716-2

**Published:** 2019-11-08

**Authors:** Ildefonso M. De la Fuente, Carlos Bringas, Iker Malaina, Benjamin Regner, Alberto Pérez-Samartín, María Dolores Boyano, María Fedetz, José I. López, Gorka Pérez-Yarza, Jesus M. Cortes, Terrence Sejnowski

**Affiliations:** 1 Department of Nutrition, CEBAS-CSIC Institute, Espinardo University Campus, Murcia, 30100 Spain; 2 Department of Mathematics, Faculty of Science and Technology, University of the Basque Country, UPV/EHU, Leioa, 48940 Spain; 3 Department of Cell Biology and Histology, Faculty of Medicine and Nursing, University of the Basque Country, UPV/EHU, Leioa, 48940 Spain; 4 Department of Applied Mathematics, Statistics and Operational Research, Faculty of Science and Technology, University of the Basque Country, UPV/EHU, Leioa, 48940 Spain; 5 Insight Data Science., Palo Alto, California USA; 6 Department of Neurosciences, Faculty of Medicine and Nursing, University of the Basque Country, UPV/EHU, Leioa, 48940 Spain; 7 Department of Cellular Biology and Immunology, Institute of Parasitology and Biomedicine “López-Neyra”, CSIC, Granada, 18100 Spain; 8 Department of Pathology, Cruces University Hospital, Biocruces-Bizkaia Health Research Institute, University of the Basque Country, UPV/EHU, Barakaldo, 48903 Spain; 9 Biocruces-Bizkaia Health Research Institute, Cruces University Hospital, Barakaldo, 48903 Spain; 10 IKERBASQUE: The Basque Foundation for Science, Bilbao, 48013 Spain; 11 Computational Neurobiology Laboratory, Howard Hughes Medical Institute, The Salk Institute for Biological Studies, La Jolla, California 92037 USA; 12 Division of Biological Sciences, University of California, San Diego, La Jolla, California 92093 USA

**Keywords:** Applied mathematics, Statistics

## Abstract

For a wide range of cells, from bacteria to mammals, locomotion movements are a crucial systemic behavior for cellular life. Despite its importance in a plethora of fundamental physiological processes and human pathologies, how unicellular organisms efficiently regulate their locomotion system is an unresolved question. Here, to understand the dynamic characteristics of the locomotion movements and to quantitatively study the role of the nucleus in the migration of *Amoeba proteus* we have analyzed the movement trajectories of enucleated and non-enucleated amoebas on flat two-dimensional (2D) surfaces using advanced non-linear physical-mathematical tools and computational methods. Our analysis shows that both non-enucleated and enucleated amoebas display the same kind of dynamic migration structure characterized by highly organized data sequences, super-diffusion, non-trivial long-range positive correlations, persistent dynamics with trend-reinforcing behavior, and move-step fluctuations with scale invariant properties. Our results suggest that the presence of the nucleus does not significantly affect the locomotion of amoeba in 2D environments.

## Introduction

Cellular migration is a crucial and complex systemic behavior of all cells endowed with directional motility. Free cells migrate for critical activities like locating food and avoiding predators or adverse conditions to enhance their chances for survival. In multicellular organisms, locomotion movements are essential for a plethora of fundamental physiological processes, such as embryogenesis, tissue morphogenesis, organogenesis, tissue repair and immune responses. Indeed, deregulated cellular migration is involved in many important human diseases, including immunodeficiencies and cancer^1,2^.

Although a notable progress is being made in understanding the biochemical mechanisms involved in the cellular migration, how cells efficiently regulate their locomotion through diverse environments, and move under the absence or presence of external cues, is an important unresolved issue in contemporary biology. Most cellular migration studies focus on fundamental molecular processes involved in the cellular locomotion system such as, the cytoskeleton^3,4^, adhesion complexes^5^, molecular signaling and regulatory networks^6,7^. Quantitative studies on chemotaxis have provided evidences that cell locomotion exhibits long range correlations in response to dynamic soluble gradients^8^. Other researchers have shown robust correlations between cell speed and persistence time (the time needed for a cell to change its motion direction) under the presence of external molecular guidance^9^. In the absence of external stimuli, cell migration has been described as persistent random walking^10,11^. Likewise, cell migration has been characterized by anomalous dynamics described by super-diffusion, non-Gaussian spatial probability distributions of the diffusion process and power-law decays of the velocity autocorrelations^12^. In addition, several studies have observed that certain cell locomotion patterns are consistent with Lévy walks^13,14^, although some controversy remains regarding the accuracy of Lévy flight foraging^15^.

One of the key issues in cellular migration is the role of the nucleus in the regulation of the locomotion system. The nucleus, the main cellular structure containing genetic information and gene regulatory machinery, has long been postulated to play an essential implication in migration, but this role is starting to be understood only very recently. In fact, shortly after the preliminary version of our study was deposited in *bioRxiv.org*^16^, a new work has shown that the nucleus is not essential for migration on flat two-dimensional (2D) surfaces^17^. In the work of Graham and coauthors (2018) the nucleus was removed from fibroblasts and endothelial cells. They observed that the cytoplasts i.e., cells without nuclei, correctly polarize and migrate along different 2D gradients in a similar way to intact nucleated cells showing that their migration abilities are not reliant on the presence of the nucleus. Lastly, these authors found that the presence, position, and material properties of the nucleus, fundamentally its connections with the cytoskeleton, make it an important mechanical cell component to regulate normal physical-mechanical responses during the cellular migration in 3D environments. These authors concluded that cells require the physical presence of the nucleus as a necessary component of the molecular clutch involved in regulating responses to their mechanical environment. The physical properties of the nucleus, strongly connected with the cytoskeleton, make it to play an important function allowing cellular locomotion when the environment presents mechanical limitations, as it occurs in 3D conditions^17^.

Prior to the publication of Graham *et al*. (2018), different studies have addressed the mechanical implications of the nucleus in 3D locomotion movements^18–26^.

Specifically, the physical connection between the nucleus and the cytoskeleton is essential for a broad range of cellular functions, including intracellular nuclear movement and positioning, cytoskeletal organization, cell polarization, chromatin organization, cellular mechanosensing and mechanotransduction signaling^27^. The importance of nucleo-cytoskeletal coupling has become also evident by the identification of a number of diseases resulting from important changes in nuclear mechanics^27–29^.

The work of Graham *et al*., (2018), conducted with modern techniques in biology, represents a remarkable advance in the field of cell migration^30^. However the role of the nucleus in systemic cellular responses during migration is still unknown from a strictly quantitative point of view.

Here, in order to understand the dynamic characteristics of the locomotion movements and to quantitatively study the role of the nucleus in cell migration, we have analyzed the movement trajectories of enucleated and non-enucleated *Amoeba proteus* using advanced non-linear dynamic tools rooted in statistical physics, more specifically, we have mainly used the root mean square fluctuation (rmsf), the Mean Square Displacement (MSD), the Detrended Fluctuation Analysis (DFA), and the renormalization group operator (RGO). Likewise, kinematic properties were also quantified such as the directionality ratio, the average speed and the total distance travelled. For such a purpose, we have studied the directional motility in normal amoebas and cytoplasts (amoebas enucleated by micromanipulation) on flat 2D surfaces, under starving conditions and in the absence of stimuli. Historically, it is known that amoebas enucleated by micromanipulation can stay alive for long periods, reaching up to 14 days^31^. In those experiments, the full viability of the enucleated organisms was verified by adding the nucleus again 12 days after enucleation; interestingly, some amoebas were capable to reproduce their complete physiological functions, including cellular division and developing stable cultures^31^. Note that 12 days of being enucleated is a much longer period of time than the length of the *Amoeba proteus*’ cellular cycle, which although may vary depending on the environment, it is usually about 24 hours long under controlled culture conditions^32^. Several years later, another researcher group showed, by traditional biological methods, that a wide range of physiological activities, such as cell locomotion, membrane ruffling, organelle distribution, shape formation, and pinocytosis occurred in enucleated cells with cytochalasin B^33^. However, cell migration studies were not performed in these two works^31,33^.

Our analytic study shows that both non-enucleated and enucleated amoebas display the same type of dynamic migration structure characterized by highly organized data sequences, super-diffusion, non-trivial long-range positive correlations, persistent dynamics with trend-reinforcing behavior, and move-step fluctuations with scale invariant properties. “*In vivo*” migration pathways change continuously, since all trajectories present random magnitudes that vary over time, but nevertheless these cellular stochastic movements shape a kind of dynamic migration structure whose defining characteristics are unambiguously preserved in both non-enucleated and enucleated cells.

Here, the role of the nucleus in the regulation of the cellular locomotion system is analyzed from a strictly quantitative perspective using both enucleated and non-enucleated cells, and our findings suggests that the presence of the nucleus does not significantly affect the migratory trajectories of amoeba in 2D environments.

## Results

**Figure 1:**
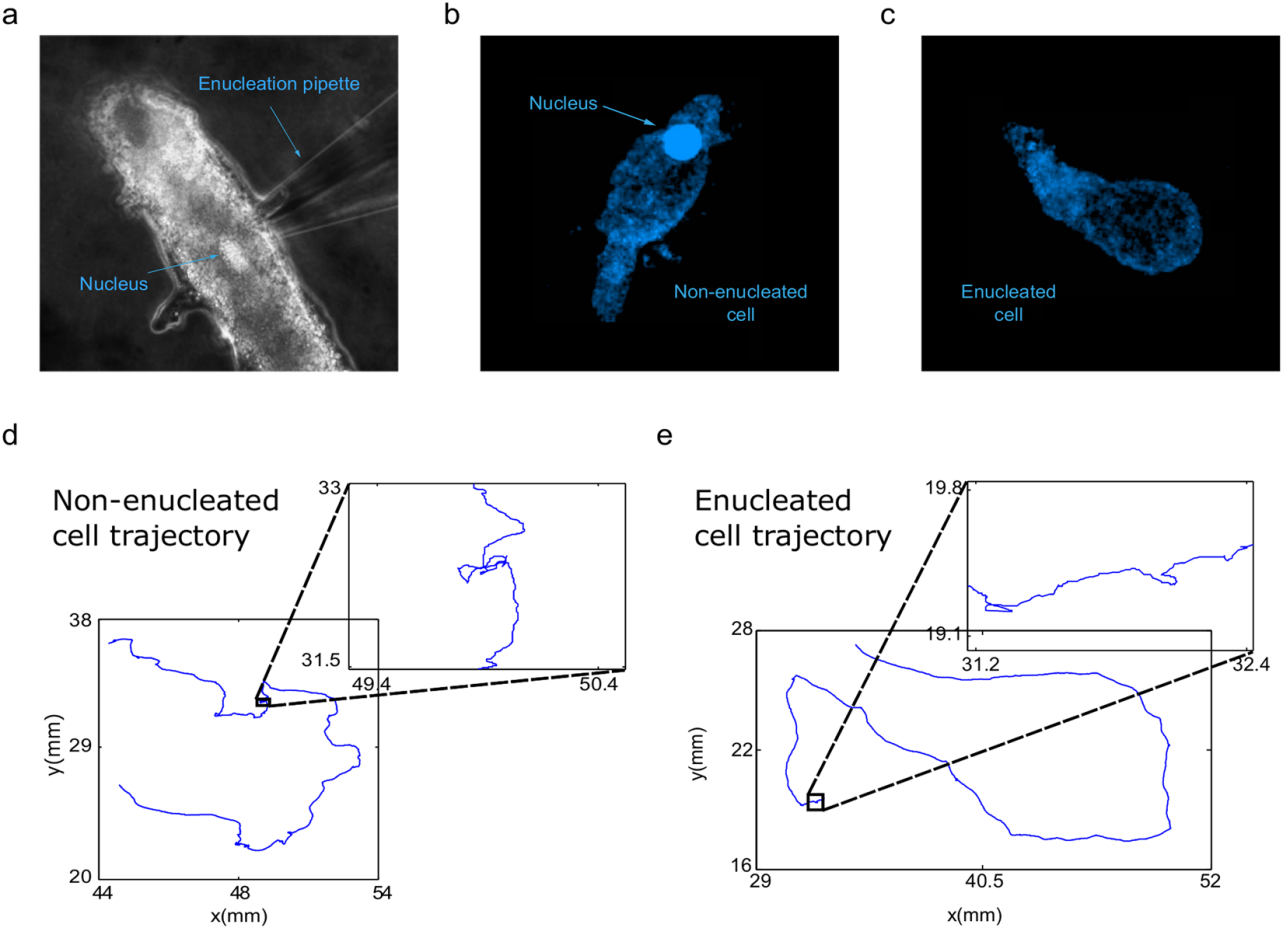
Experimental Procedures. *Amoeba proteus* cells were starved for 24 hours and half of them were enucleated using a micromanipulator: (**a**) enucleation process. The presence or absence of a nucleus was verified in the enucleated amoebas. Panels (**b**) and (**c**) illustrate fluorescent microscopy images of respectively a non-enucleated cell as a control and an enucleated cell stained by Hoechst 33258 (1 mM). All the amoebas were placed in separate nutrient-free Petri dishes, where their migration movements were recorded with a digital camera attached to a stereo microscope at a speed of one frame every two seconds: (**d**) and (**e**), digitized non-enucleated and enucleated cell trajectories, respectively. The inserts highlight local displacements by the amoeba.

Amoebas were starved for one day, after which half of the cells were enucleated by micromanipulation and finally all of them were individually placed on nutrient-free Petri dishes. The motility of each non-enucleated and enucleated cell (cytoplasts) was recorded using a digital camera attached to a stereo microscope. The digitized locomotion trajectories were analyzed in the form of time series. Once recorded the movements of each cytoplast, the absence of the nucleus was verified by Hoechst staining and fluorescence microscopy. Figure 1 summarizes the main experimental procedure, also showing two prototypical trajectories of a non-enucleated and an enucleated cell, respectively.

A preliminary observation revealed that enucleated amoebas behaved with apparent normality in relation to the substrate adhesion and overall motility. All migrations of cytoplasts and cells were characterized by multiple short move-steps, occasionally alternating with long steps and stops (Movies S1 and S2). Moreover, we also observed that cytoplasts exhibited a typical behavior for at least time periods between 2.17 h and 4.65 h (N = 20, average time = 3.72 h), during which enucleated amoebas moved with apparent normality, crept along the substrate, developed pseudopodia and phagocyted preys (see Movie S3).

**Figure 2:**
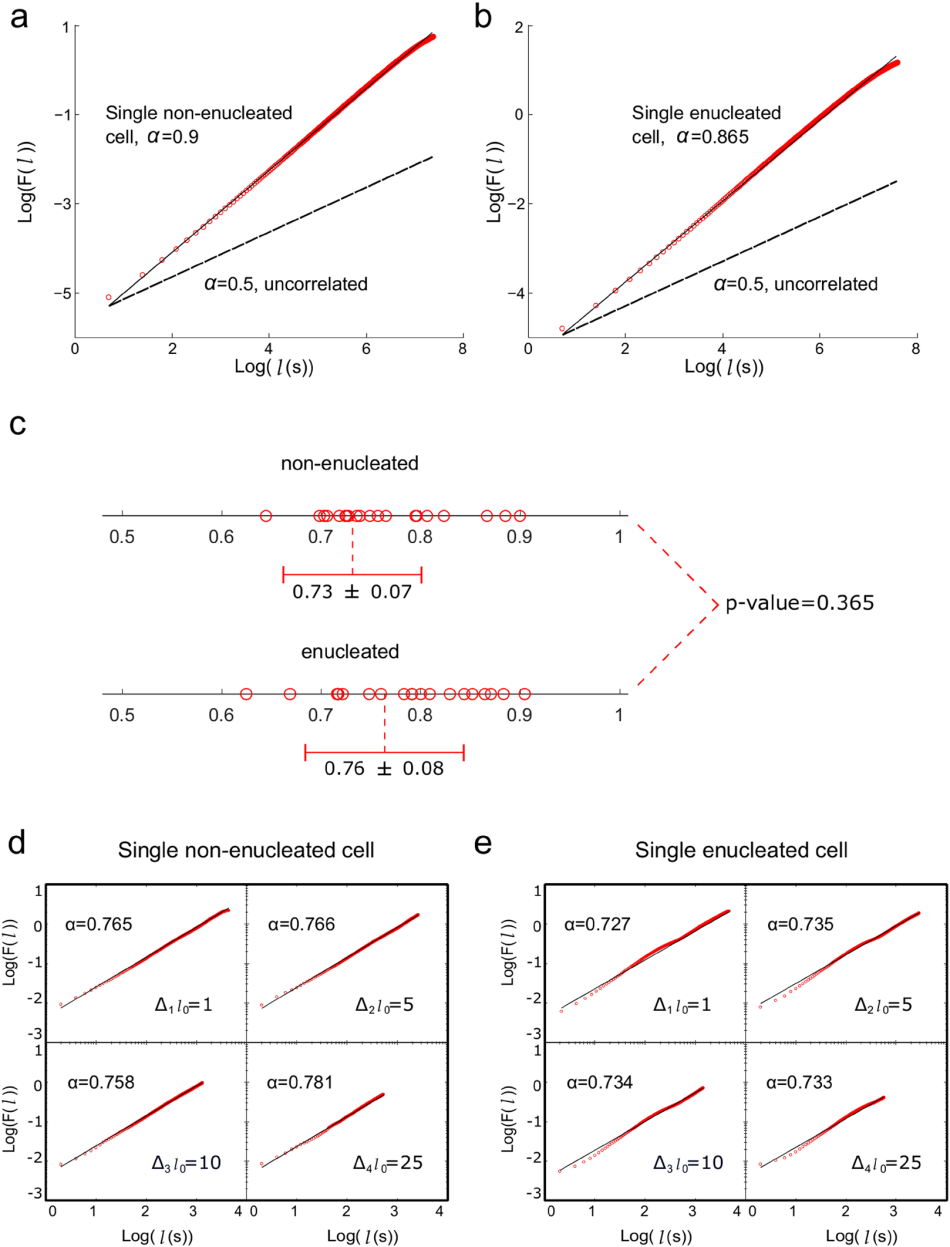
Root mean square fluctuation of the trajectories of non-enucleated and enucleated amoebas. Log-log plot of *rmsf F* versus *l* step for a prototype non-enucleated cell (**a**), and a prototype enucleated cell (**b**). The slope for the non-enucleated cell was *α* = 0.9, while the one for the enucleated was *α* = 0.865, indicating positive long-term correlations in both cases. In panel (**c**), we depict a diagram representing the values of all the scaling exponents *α* of the experimental cells separated in two groups (non-enucleated or enucleated). Average ± SD in both groups is also provided (giving a p-value after a Wilcoxon rank-sum test of 0.365). Next, we represent the analysis of the scale invariance of move-step fluctuations with respect to the increment of the step length *l*_*0*_ for a non-enucleated (**d**) and an enucleated cell (**e**), respectively. More precisely, we depict four log-log plots for each case, and calculated the scaling exponent *α* for four different increments of *l*_*0*_ (Δ*l*_0_ = 1,5,10,25), indicating undistinguishable scale-invariance properties (i.e., the exponents do not depend on the value of Δ*l*_0_) for both non-enucleated and enucleated cells.

First, we have analyzed the experimental locomotion data by applying the root mean square fluctuation (rmsf) analysis. This quantitative approach (Fig. 2a–c) allows to determine the existence of correlations in the cellular move-step fluctuations. The obtained results showed that both non-enucleated and enucleated cells display migration trajectories characterized by non-trivial long-range positive correlations. All the values of the rmsf analysis are depicted in the Tables 1 and 2. Specifically we found that the scaling exponent *α*of the rmsf exhibited an average ± *SD* equal to 0.764 ± 0.067 for non-enucleated cells, and 0.785 ± 0.075 for enucleated cells. The Wilcoxon rank-sum test showed no significant differences between the two groups (p-value = 0.365).

**Table 1:** Different trajectory metrics for non-enucleated amoebas.

Cell number	*α*	M	*γ*	*β*(*DC*)	*β*(RGO)	DR	AS	TD
*NE 1*	0.765	1,900	0.847	1.891	1.855	0.194	0.005	39.20
*NE 2*	0.824	400	0.974	1.656	1.793	0.513	0.003	39.38
*NE 3*	0.726	2,000	0.699	1.572	1.806	0.358	0.006	42.92
*NE 4*	0.795	2,100	0.686	1.440	1.859	0.082	0.005	38.75
*NE 5*	0.736	3,000	0.750	1.756	1.816	0.500	0.003	21.95
*NE 6*	0.886	300	0.981	1.597	1.723	0.155	0.003	35.61
*NE 7*	0.725	200	0.777	1.867	1.881	0.367	0.005	42.96
*NE 8*	0.699	1,100	0.657	1.533	1.776	0.117	0.003	23.06
*NE 9*	0.703	700	0.966	1.622	1.671	0.207	0.003	28.19
*NE 1*0	0.719	400	0.831	1.878	1.818	0.168	0.005	35.68
*NE 11*	0.706	1,300	0.597	1.921	1.775	0.140	0.005	42.17
*NE 12*	0.757	1,600	0.985	1.815	1.854	0.152	0.005	42.21
*NE 13*	0.900	800	0.980	1.849	1.786	0.220	0.004	32.74
*NE 14*	0.796	700	0.819	1.841	1.823	0.312	0.004	27.82
*NE 15*	0.728	800	0.958	1.705	1.880	0.717	0.004	34.67
*NE 16*	0.807	800	0.967	1.616	1.884	0.624	0.005	43.33
*NE 17*	0.749	200	0.867	1.904	1.782	0.314	0.005	24.24
*NE 18*	0.645	1,200	0.714	1.840	1.866	0.246	0.004	23.13
*NE 19*	0.739	2,400	0.825	1.941	1.911	0.135	0.006	35.43
*NE 2*0	0.867	600	0.991	1.711	1.735	0.339	0.003	47.33

NE: Non-enucleated. *α*: Scaling exponent for the rms fluctuation analysis. *M*: Long-range correlations duration. *γ*: Scaling exponent for DFA. *β*(*DC*): Scaling exponent for the MSD analysis by direct calculation (DC). *β*(*RGO*): Scaling exponent for the MSD analysis by the renormalization group operator (RGO). DR: Directionality ratio. AS: Average speed measured mm/s. TD: Total distance travelled measured in mm.

**Table 2:** Different trajectory metrics for enucleated amoebas.

Cell number	*α*	M	*γ*	*β*(*DC*)	*β*(RGO)	DR	AS	TD
*E 1*	0.625	200	0.638	1.851	1.840	0.836	0.006	43.05
*E 2*	0.865	1,000	0.993	1.767	1.764	0.452	0.003	38.30
*E 3*	0.905	2,100	0.910	1.493	1.779	0.130	0.004	29.26
*E 4*	0.792	3,100	0.993	1.727	1.790	0.184	0.004	47.60
*E 5*	0.810	500	0.969	1.748	1.803	0.472	0.005	44.70
*E 6*	0.884	1,400	0.901	1.854	1.556	0.408	0.003	42.41
*E 7*	0.784	900	0.896	1.701	1.739	0.136	0.004	28.82
*E 8*	0.761	500	0.784	1.714	1.841	0.536	0.005	37.14
*E 9*	0.871	400	0.884	1.511	1.697	0.417	0.002	43.57
*E 10*	0.830	1,800	0.951	1.608	1.550	0.415	0.002	24.31
*E 11*	0.801	300	0.954	1.507	1.586	0.427	0.002	17.16
*E 12*	0.722	2,400	0.807	1.758	1.609	0.123	0.003	24.15
*E 13*	0.718	2,400	0.921	1.520	1.326	0.253	0.002	27.34
*E 14*	0.749	300	0.952	1.562	1.800	0.416	0.004	14.28
*E 15*	0.792	1,700	0.847	1.822	1.792	0.123	0.005	28.06
*E 16*	0.717	3,100	0.998	1.506	1.774	0.309	0.005	44.30
*E 17*	0.852	200	0.610	1.697	1.845	0.063	0.005	46.05
*E 18*	0.844	2,400	0.860	1.793	1.574	0.404	0.002	36.01
*E 19*	0.716	2,400	0.917	1.760	1.870	0.243	0.005	25.84
*E 20*	0.669	200	0.721	1.667	1.854	0.294	0.005	41.72

E: Enucleated. *α*: Scaling exponent for the rms fluctuation analysis. *M*: Long-range correlations duration. *γ*: Scaling exponent for DFA. *β*(*DC*): Scaling exponent for the MSD analysis by direct calculation (DC). *β*(*RGO*): Scaling exponent for the MSD analysis by the renormalization group operator (RGO). DR: Directionality ratio. AS: Average speed measured mm/s. TD: Total distance travelled measured in mm.

As a consequence of the rmsf analysis, we also calculated the long range correlation duration (for more details, see Methods Section and Tables 1 and 2) and in all the analyzed cells and cytoplasts we found long-range correlations over periods of about 41.5 minutes on average, which corresponded to a pattern with strong dependences of the past movements lasting approximately 1, 245 move-steps. In particular, enucleated cells preserved long-range correlations up to an average duration of 45 minutes. Therefore, each cellular move-step at a given point is strongly influenced by its previous steps. This fact represents a key characteristic of the dynamic movements during the migration. The Wilcoxon rank-sum test showed no significant differences between the quantitative rmsf analysis of the two groups, non-enucleated and enucleated cells (p-value = 0.625). In addition, we have observed that the move-step fluctuations of amoebae presented scale invariance properties with respect to the increment of the step length (Fig. 1d,e).

**Figure 3:**
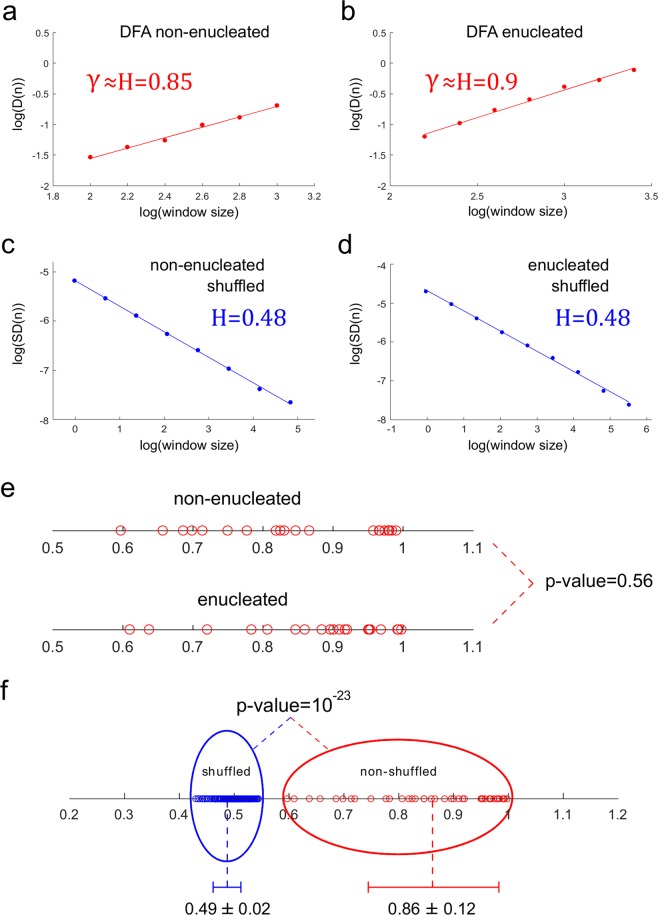
DFA and Dispersion Analysis of the trajectories of non-enucleated and enucleated amoebas. Log-log plot of the detrended fluctuation parameter *D(n)* versus window size *n* for a prototype non-enucleated cell (**a**), and a prototype enucleated cell (**b**). The scaling exponent *γ* was *γ* = 0.85 for the non-enucleated cell and *γ* = 0.9 for the enucleated cell, indicating strong long-term correlations in both cases. In panels (c) and (d), we calculate the Hurst exponent for shuffled time series, non-enucleated (H = 0.48) and enucleated (H = 0.48) respectively. Panel (e) shows a diagram with all values of exponents *γ* in all cells, separated for each group non-enucleated/enucleated, with a corresponding p-value = 0.56 after a Wilcoxon rank-sum test. Finally, in panel (f) we depict in blue 200 values of Hurst exponent corresponding to 200 shuffled time series (*H* = 0.49 ± 0.02), together with the exponents corresponding to all non-shuffled situations (*H* = 0.86 ± 0.12), depicted in red. Notice that after shuffling, the long-term correlation structure disappears (p-value = 10^−23^) for all the experimental migratory movements.

The presence of long-range positive correlations in the locomotion movements of cells and cytoplasts was validated by an alternative approach, the Detrended Fluctuation Analysis (Fig. 3a,b, Tables 1 and 2). Specifically, with this analysis we have found long-term correlations in all the experimental trajectories (the DFA scaling parameter *γ*showed an average of 0.84 ± 0.128 for the non-enucleated cells and 0.88 ± 0.112 for the enucleated cells). No significant differences were found between the two groups after the Wilcoxon rank-sum test (p-value = 0.561). DFA also allowed us to unveil that the migration movements of cells and cytoplasts exhibited a trend-reinforcing behavior i.e., if the locomotion trajectories present a decreasing trend in the past, it usually implies a decreasing tendency in the future and vice versa, an increase in a set of the move-step values in the past is likely to be followed by an increasing trend in the future (Methods Section). The high reliability of DFA analysis was tested by applying a shuffling procedure (4,000 shuffled time series in total), showing that the high correlation values measured from the experimental migration series disappeared after shuffling (see for more details Fig. 3c–e). This fact confirms that a complex structure characterized by highly organized move-step sequences underlies in the migration trajectories of the two groups (non-enucleated cells and cytoplasts), and also indicates that the dynamic structure of highly organized data sequences observed in all the move-steps trajectories could not be found by chance (p-value = 10^−23^).

**Figure 4:**
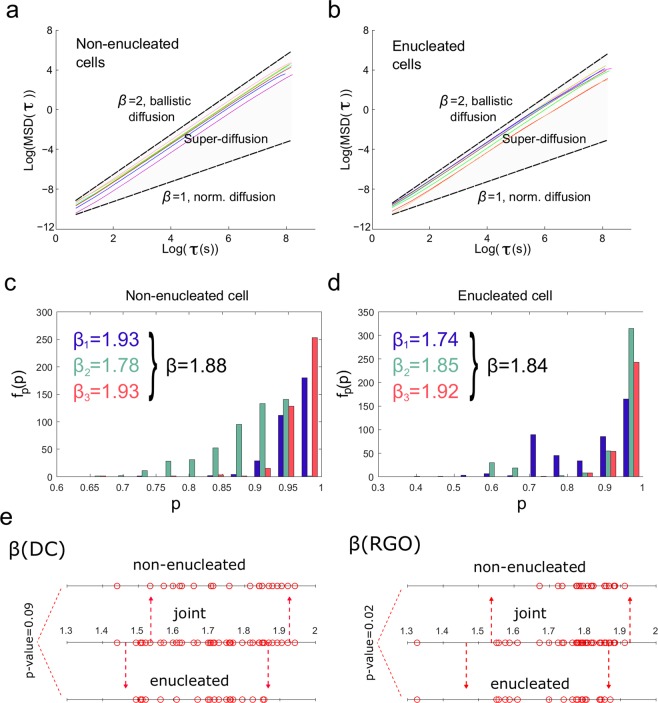
Mean Square Displacement of trajectories of non-enucleated and enucleated amoebas. Panels (a) and (b) show the direct calculation (DC) of the exponent *β* by fitting log-log plots of MSD as a function of the time interval *τ*, for 8 prototypic non-enucleated and 8 enucleated cells, respectively. *β* = 1 indicates normal diffusion while *β* = 2 indicates ballistic diffusion. The grey region defines the area of super-diffusion, within which all experimental values are contained in. The fact that τ_max_ = 1/4th of the data length, implies that super-diffusion last for long scales. In panels (c) and (d), we show results of the calculation of the exponent *β* by using the RGO method, after fitting the bar plots of the frequency of each probability (*f*_*p*_(*p*)) as a function of the probability *p*. The resulting *β* are obtained by averaging the *β*_*i*_ exponents obtained for each *i*-th block of 1,000 time points (colored in blue for *β*_1_, in green for *β*_2_ and in red for *β*_3_). Finally, panel (e) illustrates a diagram representing the values of all values of the *β* exponents for all cells, for both methods (DC or RGO) and three different cases (non-enucleated, enucleated and joint – all cells together –). We also provide p-values for all different comparisons.

To quantify the amount of space explored by the amoebas during their locomotion, we calculated the Mean Square Displacement (MSD). In this analysis, the variable *β* which characterizes the behavior of the diffusion process showed an average 1.748 ± 0.148 for the non-enucleated cells and 1.678 ± 0.123 for the enucleated cells (Fig. 4a,b, and Tables 1 and 2). These values correspond to a super-diffusion process, a complex behavior with a high non-linear relationship to time, which seems to govern both types of cell trajectories. Super-diffusion also suggested an efficient systemic movement to localize nourishment^34,35^. No significant differences were found between the two groups after the Wilcoxon rank-sum test in the variable *β* (p-value = 0.093). In addition, the correctness of the super-diffusion trajectories in cells and cytoplasts was also validated by an alternative approach, the renormalization group operator (RGO) (see for more details Fig. 4c–e, Tables 1 and 2, and Methods Section).

**Figure 5:**
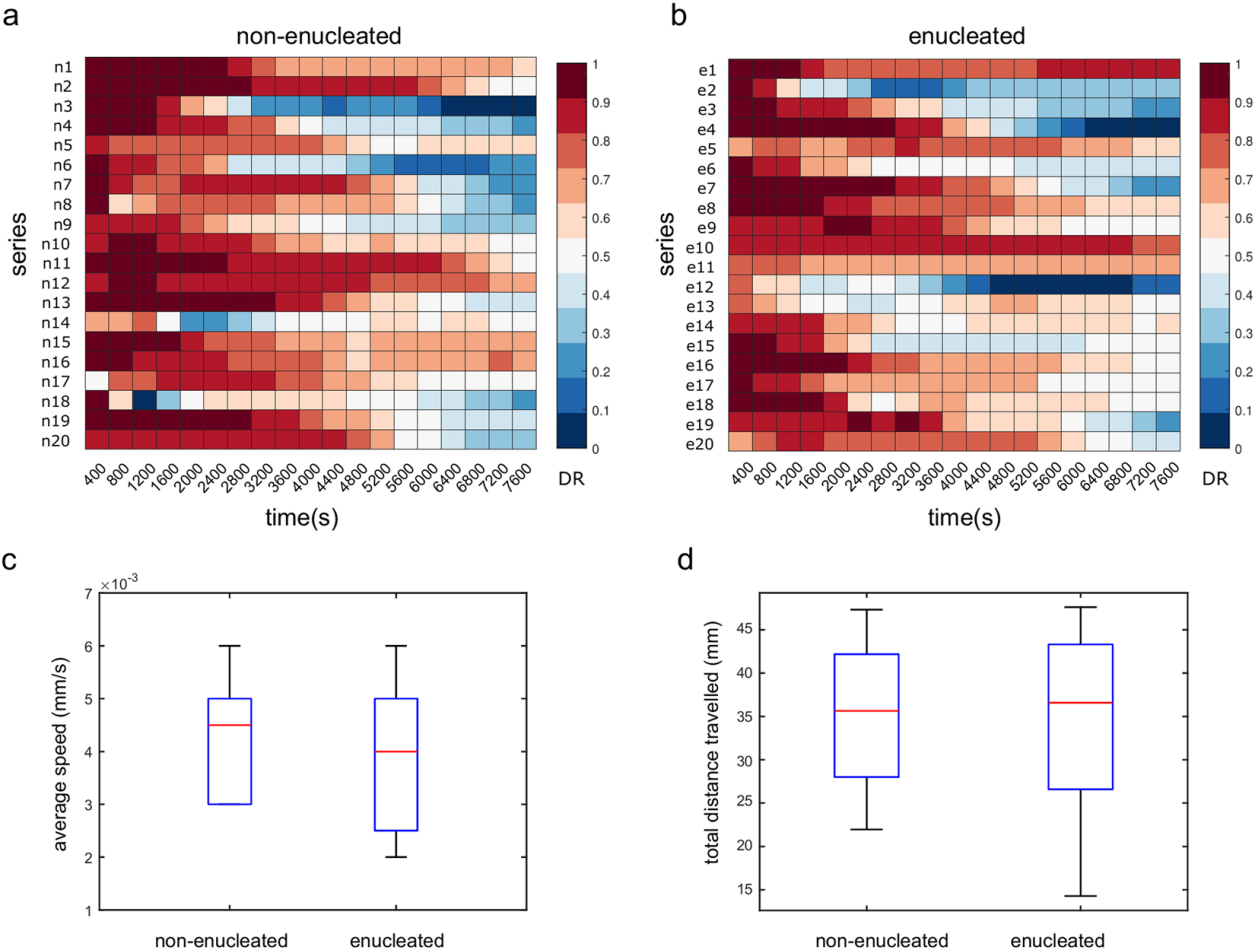
Directionality Ratio, Average Speed and Total Distance travelled of the trajectories of non-enucleated and enucleated cells. In panels (a) and (b), we depict *heatmaps* for the Directionality Ratio, with values varying from 0 (in blue) and 1 (red). Each row in both panels corresponds to a single cell, while in columns we represent the endpoint of the DR (which was increased every 400 seconds). No significant differences were found for DR between the two groups (p-value = 0.525), non-enucleated (panel a) and enucleated (panel b). In panels (c) and (d), we represent a boxplots of the distributions of average speed and total distance travelled for both non-enucleated and enucleated cells, where no significant differences were found between the two groups (the respective p-values were 0.156 and 0.818). Therefore, the three metrics directionally ratio, average speed, and total distance travelled show similar migration characteristics for both non-enucleated and enucleated cells.

Likewise, to quantify some kinematic properties of the cell migration trajectories, we studied the directionality ratio (DR), the average speed (AS), and the total distance travelled (TD) of amoebas. DR analysis quantifies the trajectory straightness, ranging between 0 (for fully curved trajectories) and 1 (for fully straight trajectories). This statistics was calculated in two different scenarios, in the first one, we analyzed the DR globally, by considering only the start and end point of the trajectory which provided values ranging between 0.082 and 0.717 (average 0.293 ± 0.177) for the non-enucleated cells (Table 1), and between 0.063 and 0.836 (average 0.332 ± 0.185) for the enucleated cells (Table 2). In this case, the Wilcoxon rank-sum test suggested that there were no differences in DR values (p-value = 0.508). In the second scenario, we calculated the DR for several endpoints, starting with 200 time points (i.e, 400 seconds of trajectory), and increasing it by periods of 200 time points, until reaching 3,800 time points (or equivalently, 7,600 seconds), which gave 19 values of DR for each cell. Once again, no significant differences were found between the DR values of non-enucleated and enucleated cells (p-value = 0.525). This second study is illustrated in Fig. 5a,b where a *heatmap* with colors associated to each DR value is depicted (colors varied from blue to red for values close to 0 and 1, respectively).

Next, we calculated the average speed (AS) of the trajectories, which ranged between 0.003 and 0.006 mm/s (average 0.004 ± 0.001) for the non-enucleated cells (Table 1 and Fig. 5c) and between 0.002 and 0.006 mm/s (average 0.004 ± 0.001) for the enucleated cells (Table 2 and Fig. 5c). No differences in AS were found after the Wilcoxon rank-sum test (p-value = 0.156).

Finally, we compared the total distance travelled (TD) by each cell in the same time (we studied this magnitude during the first 130 minutes, which is the duration of the shortest trajectory). The distance obtained ranged between 21.95 and 47.33 mm (average 35.04 ± 7.87) for the non-enucleated cells (Table 1 and Fig. 5d) and between 14.28 and 47.6 mm (average 34.2 ± 10.12) for the enucleated cells (Table 2 and Fig. 5d). No significant differences were found between the two groups after the Wilcoxon rank-sum test (p-value = 0.818). Hence, these three metrics, DR, AS and TD, show that cells and cytoplasts presented similar kinematic properties.

**Figure 6:**
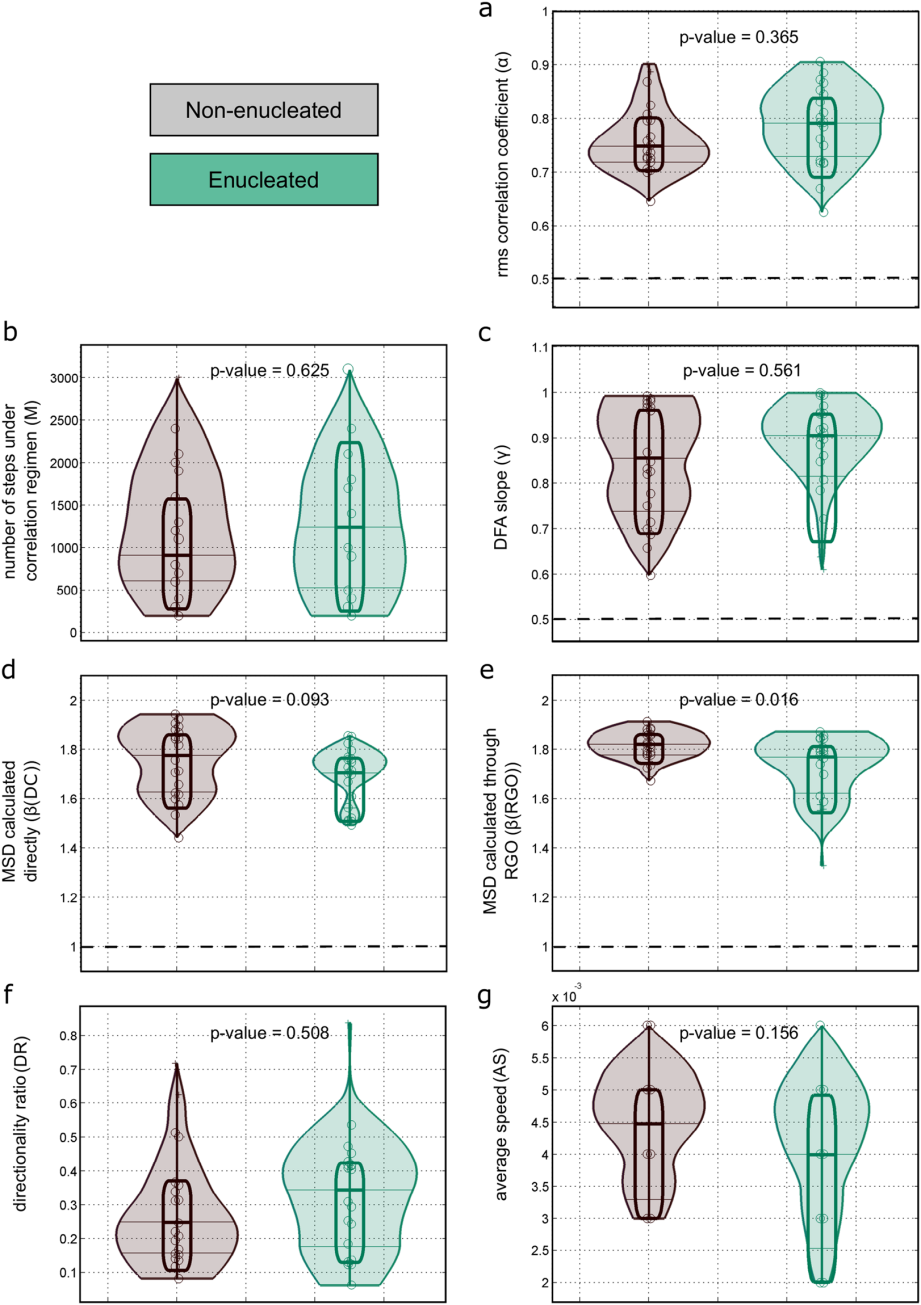
Different non-liner metrics show undistinguishable characteristics in the migration trajectories between non-enucleated and enucleated cells. We illustrate p-values and distributions for the most relevant results in our study. Violin plots include similar information as the one in boxplots, but in addition, they also incorporate the probability distribution for the values of the different metrics and groups (colored in gray-brown for non-enucleated cells and in green for enucleated cells). Panel (a), rmsf correlation coefficients *α*. Panel (b), number of steps within correlation regime M. Panel (c), DFA slopes *γ*. Panel (d), *β* values of MSD obtained by the direct calculation method. Panel (e), *β* values of the MSD obtained by the RGO method. Panel (f), global directionality ratio DR. Panel verage speed AS. From all these metrics, it can be concluded that both enucleated and non-enucleated cells have similar underlying properties in their migration movements.

Last, a violin graph (Fig. 6) depicts the most relevant results of our quantitative analysis. This Figure show the p-values and the distributions of the rmsf correlation coefficients *α* (Fig. 6a), the number of steps under correlation regimen M (Fig. 6b), the DFA slopes *γ* (Fig. 6c), the *β* values of the MSD calculated directly (Fig. 6d), the *β* values of the MSD calculated through RGO (Fig. 6e), the global directionality ratios DR (Fig. 6f), and the average speeds AS (Fig. 6g). Strikingly, all metrics show that both enucleated and non-enucleated cells have similar properties in their migration movements.

## Discussion

For a wide range of single cells, from prokaryotes to eukaryotes, the ability to perform controlled migration movements from one location to another is a crucial complex systemic feature of cellular life. However, despite its importance in a plethora of fundamental cellular processes, how unicellular organisms efficiently regulate their locomotion system is an unresolved issue in current biology.

Here, to understand the dynamic characteristics of the locomotion movements and to quantitatively study the role of the nucleus in the migration we have analyzed the movement trajectories of enucleated and non-enucleated *Amoeba proteus* using advanced non-linear dynamic tools rooted in statistical physics. All our experimental migration trajectories were obtained under starving conditions, on flat two-dimensional surfaces and in the absence of external stimuli.

To summarize, our quantitative analysis shows that both cells and cytoplasts display a kind of dynamic migration structure characterized by highly organized data sequences, super-diffusion, non-trivial long-range positive correlations, persistent dynamics with trend-reinforcing behavior, and move-step fluctuations with scale invariant properties. The locomotion movements of cells and cytoplasts change continuously, since all trajectories present random magnitudes that vary over time, but nevertheless these cellular stochastic movements shape a dynamic migration structure whose defining characteristics are preserved in both non-nucleated and enucleated cells. Such dynamic migration structure characterizes the way in which the locomotion movements occur.

In a previous work with non-enucleated cells, it has been shown that cell migration is characterized by anomalous dynamics described by super-diffusion, non-Gaussian spatial probability distributions of the diffusion process and power-law decays of the velocity autocorrelations^12^. To note, these authors found a gradient of different super-diffusion processes, with maximum values of *β* = 1.8, similar to our work.

It is well known that the nucleus plays an important role in cell migration in three-dimension spaces (3D). The presence, position, and material properties of the nucleus, fundamentally its connections with the cytoskeleton, make it an important cell component to regulate normal physical-mechanical responses during the cellular migration in 3D environments^17–22^. In particular, the physical role of the nucleus in 3D cell migration has been recently reviewed paying special attention to the mechanisms of cell motility, the mechanical properties of the nucleus, the nuclear positioning and cell polarization, the nucleus-cytoskeleton connection-dependent migration, and other key issues^23^.

While in 3D cells require the physical presence of the nucleus as a necessary component of the molecular clutch, involved in regulating responses to their mechanical environment^30^, our results show that enucleated amoebas preserved the dynamic properties in their migration movements same as the non-enucleated cells. This fact suggests that the nucleus has a minor role in regulation of the migration movements of amoebas in 2D environments. This conclusion, obtained in a quantitative way, agrees with the recent results reported by Graham and coauthors (2018) using biological techniques on flat 2D surfaces. More precisely, they removed the nucleus from fibroblasts and endothelial cells and observed that the cytoplasts correctly polarize and migrate along different 2D gradients in a similar way to intact nucleated cells showing that their migration abilities did not depend on the presence of the nucleus^17,30^.

Our study is the final result of a collaborative work using quantitative approaches whose preliminary results were deposited in Biorxiv in 2017^16^ and represented the first evidence proving that cell migration in 2D surfaces is not directly controlled by the nucleus.

The properties of the dynamic migration structure here analyzed seem to be an intrinsic characteristic of the physiological processes belonging to the cellular locomotion system. In general, cellular migration is controlled by complex molecular and metabolic networks^3,4,6^, which operate as non-linear systems far from equilibrium^36^. These biochemical networks shape an intricate interplay of multiple components of the cell migration machinery, including the actin cytoskeleton, ion channels, adhesion complexes, transporters, signaling molecules and regulatory proteins such as the Arp2/3 complex or the ADF/cofilin family proteins^37–39^. Such locomotor apparatus behaves as a complex dynamic system from which emerges some systemic properties detected here. As a consequence of the self-regulatory activity driven by the metabolic and molecular processes evolved in the locomotion system, each amoeba and cytoplast has the ability to orientate efficiently its migration movement even when no information exists about where the food is located.

## Methods

### Experimental model

We have analyzed the trajectories of 40 *Amoeba proteus* in the absence of external cues, 20 of which had been enucleated by micromanipulation. All amoebas were starved for one day at the beginning of the experiments, after which, half of the cells were enucleated and all of them were individually placed on nutrient-free Petri dishes. The motility of each cytoplast and cell was recorded using a digital camera attached to a stereo microscope, acquiring images every 2 seconds over a period between t_min_ = 130 min and t_max_ = 279 min (average = 223 min). The digitized locomotion trajectories were analyzed in the form of time series using non-linear dynamic tools. Finally, after recording the movement of each cytoplast, the absence of a nucleus was verified by Hoechst staining and fluorescence microscopy (see Extended Data Fig. 1).

### Enucleation

Each amoeba was twice washed in a Simplified Chalkley’s medium and after, half of them, enucleated on standard Petri dishes using a Sutter MP-225 micromanipulator. For enucleation, a thin glass pipette was introduced in the amoeba’s cytoplasm through the cell membrane and the nucleus was then manually sucked out. It is necessary to state that the enucleation process is quite aggressive and causes an important injury on the cell’s membrane as well as extracts a portion of the cytoplasm together with the nucleus. Therefore, the technique was attempted a maximum of two times per cell. Once enucleated, the cytoplasts were left undisturbed for about 15 minutes.

### Track recording, digitizing and significance

Each enucleated and non-enucleated amoeba was individually placed on a single nutrient-free Petri dish, where the motility of each cell was recorded using a digital camera attached to a SM-2T stereomicroscope. The size of the observation field was of approximately 7 × 5 cm and images were acquired every 2 seconds, over a period between 130 (3,900 frames) and 279 (8,370 frames) minutes long, with average of 223 minutes (6,690 frames). The duration depended on whether the amoeba was staying within the vision field and not moving outside. Inclusion criterion was based on only considering trajectories that lasted at least 2 hours within the field of vision. We performed manual tracking using the TrackMate software in ImageJ (http://fiji.sc/TrackMate), as automated tracking software is often inaccurate^40^. Each track corresponded to a different amoeba, and a single cell was never recorded more than once. 40 digitized trajectories, divided into two equally sized groups (enucleated and non-enucleated) were analyzed.

### Cell staining for nucleus removal verification

For the group of enucleated cells, the nucleus removal was verified by Hoechst staining and fluorescence microscopy. The cells were fixed in paraformaldehyde (4%) for 5 minutes and then permeabilized in Triton-X 100 (0.1%) for 5 minutes. Subsequently, the amoebas were stained with Hoechst 33258 (1 mM) for 10 minutes and finally observed under an Olympus inverted fluorescence microscope (High Resolution and Analytic Microscopy, SGIker, UPV/EHU).

### Root mean square fluctuation (rmsf) analysis

The rmsf analysis is a classical method in Statistical Mechanics based on the ideas raised by Gibbs^41^ and Einstein^42^, later developed and utilized to quantify physiological signals^43,44^. We applied the rmsf method to assess the presence of long-range correlations in the move-step fluctuations time-series, following the Viswanathan’s procedure^45^.

To quantitatively characterize the movements of the cells (Fig. 2), we analyzed the scaling behavior of the relative fluctuation along their trajectories (the deviation of the move-step length from its average) by applying the rmsf method, an approach used for the precise determination of long-range correlations in the time series of move-step fluctuations. In short, if we represent the move-step time series as *u*(*t*) = *u*(1),*u*(2), …, *u*(*t*_*max*_), we can define the net displacement after *l*steps as

1 $$y(l)\equiv \mathop{\sum }\limits_{i=1}^{l}\,{\rm{u}}({\rm{i}}),$$

and the rmsf as

2 $$F(l)\equiv \sqrt{ < \Delta y{({\rm{l}})}^{2}\, > - < \,\Delta y({\rm{l}}){ > }^{2}},$$

where Δ*y*(*l*) ≡ *y*(*l* + *l*_0_) − *y*(*l*_0_), and brackets denote the average over all possible values of *l*_0_. Long-range correlations are detected by a power-law relation such that *F*(*l*)~*l*^*α*^. For uncorrelated data, the fluctuation exponent *α* is equal to 0.5, whereas *α* > 0.5 or *α* < 0.5 indicate respectively the presence of positive or negative long-range correlations^14^.

### Long-range correlation duration (M)

As a consequence of the rms analysis, one can calculate the long-range correlation duration (M), which is defined as the number of move steps during which the regime of long-range correlations preserves, it was assessed by the maximum value of *l* at which the scaling *F*(*l*) started to curve in a log-log representation (Fig. 2).

### Scaling invariance

In addition, we have studied the scaling invariance in these movements by taking different increments of *l*_0_ and calculating the exponent *α*. For this analysis, instead of averaging all the possible *l*_0_ when calculating *F*(*l*), we took *l*_0_ with four different increments (Δ_1_*l*_0_ = 1, Δ_2_*l*_0_ = 5, Δ_3_*l*_0_ = 10, Δ_4_*l*_0_ = 25) and calculated their scaling exponent *α*. The values obtained in the four scenarios were very similar, indicating that the experimental migration series presented scaling invariance with respect to *l*_0_ in the rmsf calculation. This behavior is depicted in Fig. 1d,e, where we illustrate the scaling exponent calculated for the four *l*_0_ increments for a prototype experimental non-enucleated and enucleated cell, respectively. Therefore, this analysis indicated that the move-step fluctuations of all amoebas presented scale invariance properties with respect to the increment of the step length (Fig. 2).

### Detrended fluctuation Analysis (DFA)

DFA is a method proposed by Peng and coauthors to detect long-range correlations in time series^46^, widely used to quantify physiological signals^47^. Given a trajectory time series *u*(*t*), we first obtained the signal profile by computing the cumulative sum of the series as

3 $$z(t)=\mathop{\sum }\limits_{k=1}^{t\,}\,(u(k)- < \,u\, > \,),$$

where brackets indicate the average of *u*(*k)*. The time series *z*(*t*)is then divided into boxes of equal length *n*, and the local trend *z*_n_(*t*) in each box is subtracted. The fluctuation of this detrended signal is calculated by

4 $$D(n)=\sqrt{\frac{1}{{t}_{max}}\mathop{\sum }\limits_{t=1}^{{t}_{max}}\,{[z(t)-{z}_{n}(t)]}^{2}}.$$

This computation is repeated for all box sizes, obtaining a relationship between fluctuations *D* and box sizes *n*. A linear relationship on a log-log graph indicates the presence of long-range correlations, i.e., *D*(*n*)~*n*^*γ*^. In particular, the process exhibits positive long-range correlations when 0.5 < γ < 1.

Our DFA analysis (Fig. 3) showed that the scaling parameter *γ* ranged from 0.597 to 0.991 (average 0.844 ± 0.128) for the non-enucleated cells (Table 1) and from 0.61 to 0.998 (average 0.875 ± 0.112) for the enucleated cells (Table 2). All the experimental time series exhibited persistent behavior with *γ* > 0.5 being the global mean of *γ* = 0.697 ± 0.11, which indicates that the properties of long-term correlations dominate the migration trajectories of both non-enucleated and enucleated amoebas. In Fig. 3a,b, we illustrate the regression lines of a DFA process applied to examples of experimental non-enucleated and enucleated migration trajectories, which gave *γ* = 0.85 and *γ* = 0.9, respectively, indicating a strong structure of long-term correlations in both cases. In Fig. 4e, all the values of this analysis are represented, separated according to each case. Besides, no differences were found after the Wilcoxon rank-sum test in the variable *γ* (p-value = 0.561).

In order to estimate the significance of our results, we have performed a shuffling procedure that defines the null-hypothesis. Notice that after shuffling, the experimental locomotion series became Gaussian white noise, and a recommendable tool for studying the correlation structure of this kind of series is calculating the Hurst exponent by Dispersion Analysis^48,49^.

### Dispersion analysis (DA)

The Dispersion Analysis (DA) method is applied for the estimation of the Hurst exponent (H) on fractional Gaussian noise (fGn)^49^.

For different bins of length *n*, with *n* varying from 2 to *N*/2, one can define the standard deviation *SD(n*) of the series formed by the mean of the *n* consecutive values of the original series *x*_*i*_. That is, *SD(n*) is the standard deviation of the series *y*_*n,i*_, where ${y}_{n,i}=\frac{{x}_{i}+\ldots +{x}_{i+n-1}}{n}$. Now, the relation between log(*SD(n*)) and log(*n*) is approximately linear: *SD*(*n*) = *SD*(1) · *n*^*H*−1^, with slope H-1, where H is the Hurst coefficient and *SD*(1) the standard deviation calculated on the first window.

The Hurst exponent H satisfies 0 ≤ H ≤ 1. For a random process with independent increments, H is 0.5. When H differs from 0.5, the process is properly fractional and indicates the existence of long-term memory, in which future events have long-term correlations with past events. If H > 0.5, it indicates a biased random process with persistent trend-reinforcing behavior. In this case, for several previous transitions, an increment on the average value implies an increasing trend in the future. Conversely, a previously decreasing trend for a sequence of values usually implies a decrease for a similar sequence. Anti-persistent behavior is obtained for 0 ≤ H < 0.5; in this case, a previously decreasing trend implies a probable increasing trend in the future and vice versa, an increase in the past is usually followed by a decrease in the future^49^.

Since under our conditions, the scaling exponent *γ* behaves as the Hurst exponent H^50^, we have compared the scaling exponent *γ* to the H. If the original migration trajectories exhibit a correlation structure (*γ* ≈ *H* ≠ 0.5), after the shuffling such structure will disappear, thus re-applying a new Hurst analysis on the shuffled data should provide values of H close to 0.5.

According to this procedure, we performed two hundred random permutations for each experimental migration trajectory, which allowed building the null-hypothesis of no correlations. In total, we generated 4,000 random series from the experimental locomotion data. After shuffling, the results show an average Hurst exponent of 0.49 ± 0.02, indicating the absence of long-term correlations i.e., the informational structures in all shuffled locomotion series was completely lost. In Fig. 3c,d, we represent the calculation of the Hurst exponent by the Dispersion Analysis for a shuffled non-enucleated and a shuffled enucleated time series, respectively. In both cases, the Dispersion Analysis, gave H = 0.48, which indicates a breakdown of the long-term correlation structure.

In Fig. 3f, for illustrative purposes, we represent 200 of the 4,000 Hurst exponent values corresponding to 200 shuffled series (average *H* = 0.49 ± 0.02), along with the values of the original series (*H* = 0.86 ± 0.12). It can be observed that, after shuffling, the long-term correlation structure disappears completely in all the experimental migratory movements. Thus, the informational structures in all shuffled series were completely broken-down, and therefore, the correlation structure that characterizes the experimental locomotion movements could not be found by chance (p-value = 10^−23^ when the exponents of the shuffled series were compared with the experimental ones).

### Mean square displacement (*Direct calculation*)

The MSD is a method proposed by Einstein in his work concerning Brownian motion^51^, widely utilized since then, for example, to quantify cell motility^52,53^. This approach accounts for the average squared displacement in a migration trajectory over increasing time intervals or scales^48^. Specifically, the MSD is a proxy for the surface area explored by the cell over time and is related to the overall migration efficiency^34^. For a two-dimensional trajectory *P*(*t*) = [*x*(*t*),*y*(*t*)], the MSD is defined as

5 $$MSD(\tau )\equiv \frac{1}{{t}_{max}-\tau }\mathop{\sum }\limits_{t=1}^{{t}_{max}-\tau }{(r(t+\tau )-r(t))}^{2}$$

where we have defined the instantaneous modulus as $r(t)=\sqrt{{(x(t))}^{2}+{(y(t))}^{2}}$ and where *τ* denotes the time scale. Here, diffusion was studied up to a maximum time scale *τ*_*max*_ equal to 1/4th of the data size. An important property of random walks is their power law scaling, MSD*(τ*)*~*^*τβ*^, where *β* characterizes the behavior of the diffusion process. For uncorrelated Brownian motion the exponent *β* is equal to 1, when 1 < *β* < 2 holds the process is super-diffusive and when 0 < *β* < 1 is sub-diffusive. These two processes, super and sub-diffusive, encompass anomalous diffusion, and typically occur in complex systems in the presence of long-range correlations.

### Mean square displacement (Renormalization group operator)

It has recently shown that the direct calculation of the MSD can fail in the estimation of *β* for short time series^54^, an issue that can be sorted out by an alternative method based on a renormalization group operator (RGO) developed by the Nobel Prize Laureate Kenneth Wilson, who established the Theory of the Renormalization Group^55^. In short, for a random trajectory X with a set of increments I, one can define a RGO as

6 $${({{\rm{R}}}_{{\rm{n}},{\rm{p}}}{\rm{I}})}_{{\rm{i}}}\equiv \mathop{\sum }\limits_{{\rm{k}}={\rm{in}}}^{({\rm{i}}+1){\rm{n}}-1}\frac{{{\rm{I}}}_{{\rm{k}}}}{{{\rm{n}}}^{{\rm{p}}}},$$

where p > 0 and n ≥ 1. Then, a new replica trajectory J is determined as J^p,n^ = (R_n,p_I)_i_. A sequence I is called a fixed point of the RGO for a fixed p if the relationship of having equal distributions J^p,n^ = I holds for all n ≥ 1. Finally, the MSD exponent is then calculated as *β* = 2*p*. Here, following a similar algorithm as the one developed in^54^, we estimated an exponent *β*_*i*_ for each *i*-th window of 1,000 non-overlapping time points. The reported *β* is the average over all the different time windows.

The values of the exponent *β*, calculated by the RGO method, ranged between 1.671 and 1.911 (average 1.815 ± 0.061) for the non-enucleated cells (Fig. 4e and Table 1) and between 1.326 and 1.870 (average 1.719 ± 0.141) for the enucleated cells (Fig. 4e and Table 2). Therefore, both methods, the direct calculation of MSD and RGO, revealed super-diffusion in trajectories for the two types of cells. The Wilcoxon rank-sum test showed differences in the *β* value after the RGO analysis (p-value = 0.016, Fig. 4e), although super-diffusion was measured in both cases.

In order to illustrate the calculation of *β* through RGO method, in Fig. 4c,d we depicted the estimation of this statistic for a single non-enucleated and a single enucleated cell, respectively. As it is described above, for each series, the resulting *β* is obtained by averaging the *β*_*i*_ exponents obtained for each *i*-th non-overlapping block of 1,000 time points (colored in both Figs 2d and 4c in blue for *β*_1_, in green for *β*_2_ and in red for *β*_3_).

### Directionality ratio (DR), average speed (AS) and total distance travelled (TD)

Three kinematic metrics were obtained from cell trajectories (Fig. 5). The directionality ratio (DR) is a parameter that quantifies the trajectory straightness^53^, which is equal to 1 for a fully straight trajectory and equal to 0 for a fully curved trajectory. For two-dimensional trajectories given by *P*(*t*) = [*x*(*t*),*y*(*t*)], we first calculated the total trajectory length for certain *t*_*max*_as $\delta =\mathop{\sum }\limits_{t=1}^{{t}_{max}}u(t)$, where *u*(*t*) represents the displacement of the amoeba at time *t*. Next, we calculated the Euclidean distance between the start point *P*(*t*_0_) and the endpoint *P*(*t*_*max*_). The directionality ratio was then defined as *DR* = *d*/*δ*. The average speed (AS) was calculated as the average of all *u*(*t*) values divided by the time resolution (equal to 2 seconds), and the total distance travelled was calculated by summing all the displacement modules of each cell.

## Supplementary information

Digitized migration of a cytoplast

Digitized migration of a regular amoeba

enucleated amoebas moved with apparent normality, crept along the substrate, developed pseudopodia and phagocyted preys

## Data Availability

The datasets generated during and/or analyzed during the current study (video frames of original cellular trajectories, digitized videos and fluorescence images) are available in the Zenodo repository, https://zenodo.org/record/3492173#.XajMwpMzaTc.

## References

[1] Bouma G, Bums SO, Thrasher AJ (2009). Wiskott-Aldrich Syndrome: Immunodeficiency resulting from defective cell migration and impaired immunostimulatory activation. Immunobiology.

[2] Olson MF, Sahai E (2009). The actin cytoskeleton in cancer cell motility. Clin. Exp. Metastasis.

[3] Pollard TD, Borisy GG (2003). Cellular motility driven by assembly and disassembly of actin filaments. Cell.

[4] Tanaka K (2018). Structural basis for cofilin binding and actin filament disassembly. Nat. Commun..

[5] Vicente-Manzanares M, Ma X, Adelstein RS, Horwitz AR (2009). Non-muscle myosin II takes centre stage in cell adhesion and migration. Nat Rev Mol Cell Biol..

[6] Disanza A (2005). Actin polymerization machinery: the finish line of signaling networks, the starting point of cellular movement. Cell. Mol. Life. Sci..

[7] Vinogradova T, Miller PM, Kaverina I (2009). Microtubule network asymmetry in motile cells: role of Golgi-derived array. Cell Cycle.

[8] Skoge M (2014). Cellular memory in eukaryotic chemotaxis. Proc. Natl. Acad. Sci..

[9] Maiuri P (2015). Actin flows mediate a universal coupling between cell speed and cell persistence. Cell.

[10] Selmeczi D, Mosler S, Hagedorn PH, Larsen NB, Flyvbjerg H (2005). Cell motility as persistent random motion: theories from experiments. Biophys. J..

[11] Li L, Cox EC, Flyvbjerg H (2011). ‘Dicty dynamics’: Dictyostelium motility as persistent random motion. Phys. Biol..

[12] Dieterich P, Klages R, Preuss R, Schwab A (2008). Anomalous dynamics of cell migration. Proc. Natl. Acad. Sci..

[13] Ariel G (2015). Swarming bacteria migrate by Lévy Flight. Nat. Comm..

[14] Harris TH (2012). Generalized Lévy walks and the role of chemokines in migration of effector CD8+ T cells. Nature.

[15] Pyke GH (2015). Understanding movements of organisms: it’s time to abandon the Lévy foraging hypothesis. Meth. Ecol. Evol..

[16] Bringas, C. et al. Long-term memory in the migration movements of enucleated *Amoeba proteus*. *bioRxiv* 125054 (2017).

[17] Graham DM (2018). Enucleated cells reveal differential roles of the nucleus in cell migration, polarity, and mechanotransduction. J. Cell. Biol..

[18] Friedl P, Wolf K, Lammerding J (2011). Nuclear mechanics during cell migration. Curr. Opin. Cell. Biol..

[19] Petrie RJ, Yamada KM (2015). Fibroblasts lead the way: a unified view of 3d cell motility. Trends. Cell Biol..

[20] Liu L, Luo Q, Sun J, Song G (2016). Nucleus and nucleus-cytoskeleton connections in 3D cell migration. Exp. Cell. Res..

[21] Petrie RJ, Harlin HM, Korsak LIT, Yamada KM (2017). Activating the nuclear piston mechanism of 3D migration in tumor cells. J. Cell Biol..

[22] Calero-Cuenca FJ, Janota CS, Gomes ER (2018). Dealing with the nucleus during cell migration. Curr. Opin. Cell. Biol..

[23] Fruleux A, Hawkins RJ (2016). Physical role for the nucleus in cell migration. J. Phys. Condens. Matter..

[24] Lammerding J (2011). Mechanics of the nucleus. Comprehensive Physiology..

[25] Dahl KN, Ribeiro AJ, Lammerding J (2008). Nuclear shape, mechanics, and mechanotransduction. Circulation research..

[26] Dahl KN, Kahn SM, Wilson KL, Discher DE (2004). The nuclear envelope lamina network has elasticity and a compressibility limit suggestive of a molecular shock absorber. Journal of cell science..

[27] Isermann P, Lammerding J (2013). Nuclear mechanics and mechanotransduction in health and disease. Current Biology..

[28] Alvarado-Kristensson M, Rosselló CA (2019). The Biology of the Nuclear Envelope and Its Implications in Cancer Biology. International journal of molecular sciences..

[29] Davidson PM, Lammerding J (2014). Broken nuclei–lamins, nuclear mechanics, and disease. Trends in cell biology..

[30] Hawkins RJ (2018). Do migrating cells need a nucleus?. J. Cell. Biol..

[31] Ord MJ (1968). The viability of the anucleate cytoplasm of *Amoeba proteus*. J. Cell. Sci..

[32] Prescott DM (1955). Relations between cell growth and cell division. I. Reduced weight, cell volume, protein content, and nuclear volume of amoeba proteus from division to division. Exp. Cell. Res..

[33] Goldman RD, Pollack R, Hopkins NH (1973). Preservation of normal behavior by enucleated cells in culture. Proc. Natl. Acad. Sci..

[34] Faustino CL, da Silva LR, da Luz MGE, Raposo EP, Viswanathan GM (2007). Search dynamics at the edge of extinction: Anomalous diffusion as a critical survival state. Europhysics Letters.

[35] Viswanathan GM, Raposo EP, da Luz MGE (2008). Lévy flights and superdiffusion in the context of biological encounters and random searches. Physics of Life Reviews.

[36] De la Fuente IM (2015). Elements of the cellular metabolic structure. Front Mol Biosci..

[37] Artemenko Y, Lampert TJ, Devreotes PN (2014). Moving towards a paradigm: common mechanisms of chemotactic signaling in Dictyostelium and mammalian leukocytes. Cell. Mol. Life. Sci..

[38] Mogilner A, Edelstein-Keshet L (2002). Regulation of Actin Dynamics in Rapidly Moving Cells: A Quantitative Analysis. Biophys. J..

[39] Senoo H, Cai H, Wang Y, Sesaki H, Iijima M (2016). The novel RacE-binding protein GflB sharpens Ras activity at the leading edge of migrating cells. Mol. Biol. Cell..

[40] Hilsenbeck O (2016). Software tools for single-cell tracking and quantification of cellular and molecular properties. Nat. Biotech..

[41] Gibbs, J. W. *Elementary Principles in Statistical Physics Developed with Especial Reference to The Rational Foundation of Thermodynamics* (Charles Scribner’s Sons) (1902).

[42] Einstein A (1909). Zum gegenwärtigen stand des strahlungsproblems. Physikalische Zeitschrift..

[43] Ivanov PC (1999). Multifractality in human heartbeat dynamics. Nature.

[44] Ivanov PC (2001). From 1/f noise to multifractal cascades in heartbeat dynamics. Chaos..

[45] Viswanathan GM (1996). Lévy flight search patterns of wandering albatrosses. Nature.

[46] Peng CK (1994). Mosaic organization of DNA nucleotides. Phys. Rev. E..

[47] Goldberger AL (2002). Fractal dynamics in physiology: Alterations with disease and aging. Proc. Nat. Acad. Sci. USA.

[48] Eke A (2000). Physiological time series: distinguishing fractal noises from motions. Pflugers. Arch..

[49] Caccia DC, Percival DB, Cannon MJ, Raymond GM, Bassingthwaight JB (1997). Analyzing exact fractal time series: evaluating dispersional analysis and rescaled range methods. Physica A.

[50] Hardstone R (2012). Detrended fluctuation analysis: a scale-free view on neuronal oscillations. Front. Psychol..

[51] Einstein A (1905). Über die von der molekularkinetischen Theorie der Wärme geforderte Bewegung von in ruhenden Flüssigkeiten suspendierten Teilchen. Annalen der physik..

[52] Long Z (2013). Microfluidic chemostat for measuring single cell dynamics in bacteria. Lab. Chip..

[53] Gorelik R, Gautreau A (2014). Quantitative and unbiased analysis of directional persistence in cell migration. Nat. Protoc..

[54] Regner BM, Tartakovsky DM, Sejnowski TJ (2014). Identifying transport behavior of single-molecule trajectories. Biophys. J..

[55] Wilson K (1971). Renormalization group and critical phenomena II: Phase space cell analysis of critical behavior. Phys. Rev. B.

